# *Taenia martis* Cysticercosis in a Common Marmoset (*Callithrix jacchus*)

**DOI:** 10.3390/vetsci11120623

**Published:** 2024-12-04

**Authors:** Martina Bleyer, Lena Erffmeier, Olga Batura, Christian Roos

**Affiliations:** German Primate Center, Leibniz Institute for Primate Research, 37077 Göttingen, Germany; lerffmeier@dpz.eu (L.E.); obatura@dpz.eu (O.B.); croos@dpz.eu (C.R.)

**Keywords:** cestode, *Taenia martis*, common marmoset, metacestode, cysticercosis

## Abstract

*Taenia martis* cysticercosis is a rare but recently more frequently reported disease that can affect both human and non-human primates as aberrant hosts. A common marmoset was noticed as being affected by advancing weight loss that did not respond to therapy and finally had to be euthanized due to poor prognosis. A complete necropsy with gross evaluation and subsequent histological and molecular analyses was performed, revealing the presence of a cysticercosis in the thoracic and pelvic cavity and in the mesentery. Molecular analysis identified the cestode as *Taenia martis*. This is the fifth case of *Taenia martis* cysticercosis in a non-human primate and the first case in a platyrrhine primate.

## Simple Summary

A common marmoset suffered from progressive weight loss. Necropsy revealed multicystic structures in the thoracic and pelvic cavity and attached to the mesentery. Histologically, the cysts contained metacestodes identified as *Taenia martis* by molecular analysis. This is the first case of *Taenia martis* cysticercosis in a platyrrhine non-human primate.

## 1. Introduction

Cysticercosis is caused by the larval stages of taeniid cestodes. *Taenia martis* is a cestode of the Northern hemisphere, whose adult stages inhabit the small intestine of martens and other mustelid carnivores [1,2,3]. In Europe, the main definitive hosts are the stone marten (*Martes foina*) and the pine marten (*Martes martes*). Small rodents serve as the natural intermediate hosts and develop cysticerci in the pleural and peritoneal cavities after ingestion of tapeworm eggs. Primates can become aberrant intermediate hosts after oral uptake of *T. martis* eggs through contaminated food, water, or soil [2,4,5]. *Taenia martis* cysticercosis has so far been reported in seven human patients from France, the Netherlands, Switzerland, and Germany [6,7,8,9,10,11,12], emphasizing the zoonotic potential of this cestode species [1]. Among non-human primates, four cases of *T. martis* infections have been described to date, including a Tonkean macaque (*Macaca tonkeana*) from France [1], a ring-tailed lemur from Italy (*Lemur catta*) [13], a white-headed lemur from Germany (*Eulemur albifrons*) [5], and a Lac Alaotra bamboo lemur from France (*Hapalemur alaotrensis*) [2]. Etiological diagnosis was confirmed by molecular analysis in all human and non-human primate cases.

We here present the first report of *Taenia martis* cysticercosis in a common marmoset (*Callithrix jacchus*) from Germany, with reference to the clinical picture, macroscopic appearance, histological features, and molecular identification.

## 2. Case Description

Four adult male common marmosets from a private husbandry in Germany were confiscated by a German veterinary office due to poor housing conditions. Afterwards, they were handed over in the care of the German Primate Center, where they were kept according to national and international regulations regarding the keeping of non-human primates (Directive 2010/63/EU of the European Parliament and of the Council of 22 September 2010 on the protection of animals used for scientific purposes; Animal Welfare Laboratory Animal Ordinance (Tierschutz-Versuchstierverordnung) and German Animal Welfare Act (Tierschutzgesetz)). Four months after their arrival at the German Primate Center, one of the animals had to be euthanized due to a rapidly growing cystic mass in the abdomen that was histologically diagnosed as alveolar echinococcosis.

Three years later, another marmoset became clinically apparent with progressive weight loss. At this time, the animal was seven years old. Clinical examinations included blood tests, ultrasound examinations of the liver and abdominal cavity (both unremarkable), as well as bacteriological and parasitological fecal sample analysis (detection of *Giardia lamblia* with subsequent treatment). A high-energy diet was fed, but failed to stop the progressive weight loss. Finally, the marmoset was euthanized due to lack of responsiveness to therapy and a poor prognosis.

**Figure 1:**
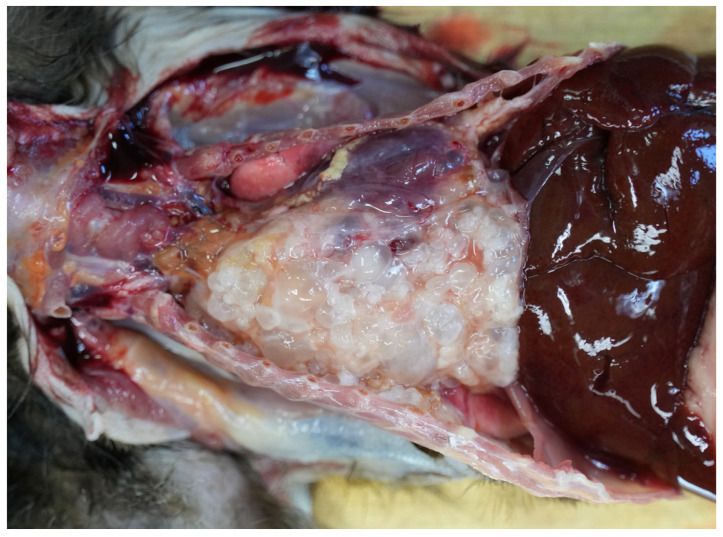
A 7-year-old male common marmoset showed a multicystic mass in the thoracic cavity consistent with metacestode tissue.

At necropsy, the marmoset was cachectic. The thoracic cavity was almost completely filled with a whitish multicystic mass that showed adhesions with the pericardium, the thoracic wall, and the pulmonary serosa (Figure 1). The diameter of cysts ranged between 2 and 10 mm. Several small cysts were also attached to the mesentery. Another multicystic mass with a size of approximately 1.2 × 1.0 × 0.8 cm was present in the pelvic cavity adjacent to the accessory sex glands.

**Figure 2:**
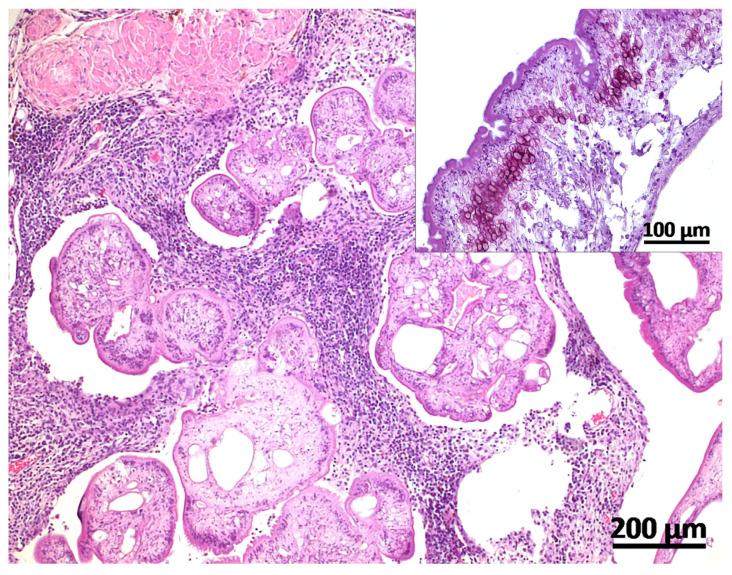
Metacestode tissue consisted of multiple cysticerci surrounded by fibrous septae with a prominent inflammatory reaction. Cysticerci had an eosinophilic tegument with a narrow muscular layer lying underneath and a loose reticular parenchyma that contained calcareous corpuscles (inset) (H&E-stained histological section, scale bar = 200 µm, inset = 100 µm).

**Figure 3:**
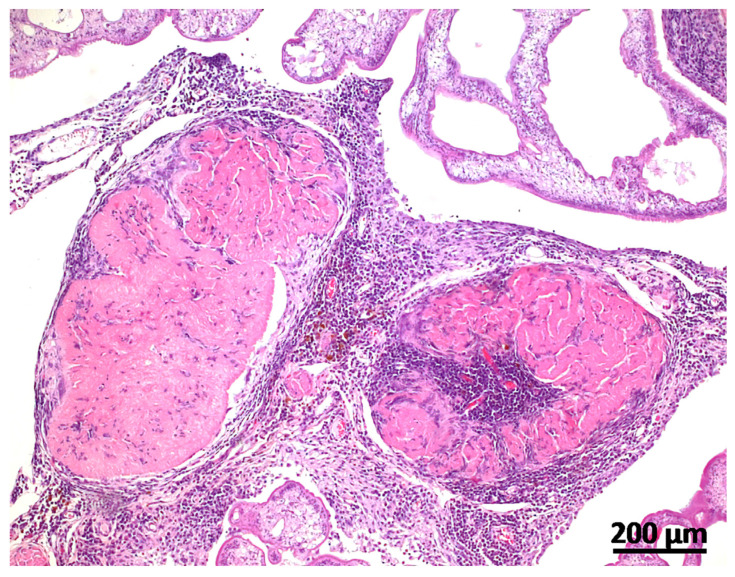
Degenerated cysticerci could be observed multifocally and presented as hyalinized material, demarcated by epithelioid macrophages and multinucleated giant cells (H&E-stained histological section, scale bar = 200 µm).

Tissue samples from a broad organ spectrum and gross lesions were placed in 10% phosphate buffered formaldehyde and were snap frozen and stored at −80 °C. Fixed tissue samples were paraffin-embedded, sectioned at 3 µm, and stained with hematoxylin and eosin (H&E). Histologically, cystic masses were composed of connective tissue septae surrounding cysticercal larvae. Within the collagenous connective tissue stroma, there were moderate to severe diffuse infiltrates of lymphocytes, plasma cells, macrophages, and fewer neutrophils and eosinophils. Siderophages and foci of dystrophic mineralization could regularly be observed. Metacestodes were characterized by an eosinophilic tegument with a narrow muscular layer lying underneath and a loose reticular parenchyma, which occasionally contained calcareous corpuscles (Figure 2). Within several sections of metacestode tissue, suckers or rostella with hooks could not be observed. Degenerated cysticerci presented as hyalinized material demarcated by epithelioid macrophages and multinucleated giant cells (Figure 3).

Extraction of DNA from suspected, snap-frozen metacestode tissue was carried out using the NucleoSpin Tissue Kit (Macherey-Nagel, Düren, Germany) following the manufacturer’s protocol. A ca. 400 bp-long fragment of the mitochondrial cytochrome c oxidase subunit 1 (cox1) was PCR-amplified using the methods outlined in Nguyen et al., 2015 [14]. The resulting PCR product was visualized on a 1% agarose gel, purified with the QIAquick PCR Purification Kit (QIAGEN, Hilden, Germany), and sent to Eurofins (Ebersberg, Germany) for Sanger sequencing using both amplification primers. Sequences were checked and corrected with 4Peaks v1.8 (nucleobytes.com (accessed on 15 January 2023)). To identify *Taenia* sp., we performed a standard BLAST search using the Basic Local Alignment Search Tool (BLAST) of the National Center of Biotechnology Information (NCBI). Moreover, we reconstructed a neighbor-joining tree in SeaView v5.04 [15] using additional orthologous sequences of other *Taenia* spp. deposited in GenBank.

**Figure 4:**
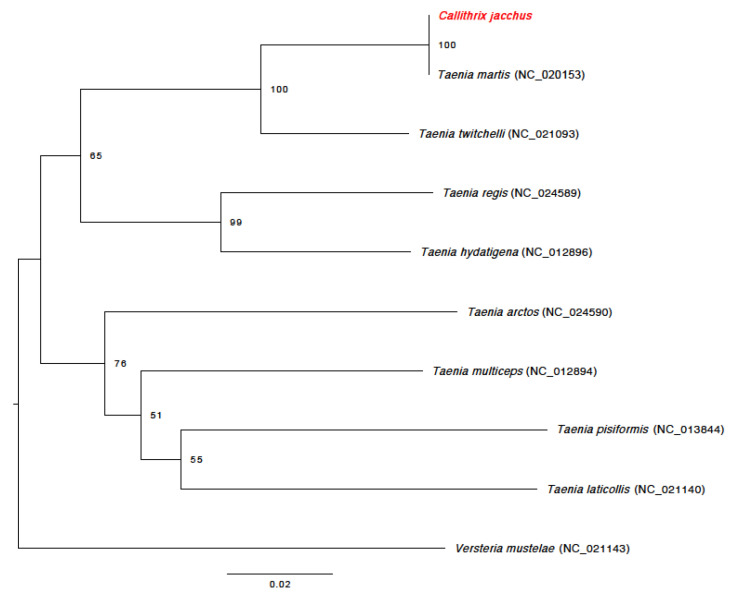
Neighbor-joining tree based on 372 bp of the mitochondrial cytochrome c oxidase subunit 1 (cox1) showing phylogenetic relationships among eight *Taenia* sp., including the sample derived from the common marmoset (highlighted in red, this study). *Versteria mustelae* was chosen as the outgroup. Numbers at nodes refer to bootstrap values and the scale below shows substitutions per site.

Comparative analyses using BLAST revealed 100% identity of the 372-bp long cox1 sequence with orthologous sequences of *T. martis* in GenBank (accession numbers NC_020153, EU544553). Similarly, the neighbor-joining tree including representatives of eight *Taenia* sp., outgroup rooted with *Versteria mustelae*, clearly depicts the *Taenia* sp. obtained from the marmoset tissue as *T. martis* (Figure 4).

## 3. Discussion

This is the fifth case of *T. martis* cysticercosis in a non-human primate and the first case in a platyrrhine non-human primate species.

In recent years, a few cases of *T. martis* cysticercosis have been reported in human and non-human primates in Germany, the Netherlands, France, Italy, and Switzerland [2,5,12]. In Europe, *T. martis* has been observed in its natural definitive (mustelids) and intermediate (rodents) hosts in Italy, Germany, The Netherlands, Belgium, Spain, Poland, Belarus, and Switzerland [6]. Prevalence rates of adult *T. martis* stages in definitive hosts show regional variations and are indicated with 5.88% in the pine marten in Spain [16], 33.3% in the stone marten in Northwest Italy [17], and 36% in the stone marten in Southwest Germany [4,13]. Data about the incidence of *T. martis* in mustelid carnivores in other parts of Germany are lacking in the literature.

Prior to confiscation, the common marmoset with *T*. *martis* cysticercosis was part of a private marmoset husbandry in Germany. Details about the exact location of the previous habitation and the keeping conditions were unfortunately not available. However, the infection with *T. martis* eggs has likely occurred prior to the relocation of the animals, because the marmoset colony of the German Primate Center lives in solely indoor enclosures, and no previous cases of cysticercosis have been observed in over 30 years. The fact that a second animal of the confiscated marmoset group was diagnosed with a larval cestode infection shortly after transfer to the German Primate Center also supports the assumption of an infection in the previous husbandry.

Among the reported non-human primate cases of *T. martis* cysticercosis, the Lac Alaotra bamboo lemur from France originated from Poland and had only spent three months at a French zoo before its death [2]. Thus, the infection most likely occurred in Poland. The ring-tailed lemur from an Italian zoo was transferred from the Czech Republic approximately six years before it died. In this case, it remains questionable where the initial exposure to *T. martis* eggs occurred [13].

Although details about the housing conditions of the marmosets in the previous husbandry are lacking, it can be suspected that the animals came into contact with soil, grass, or food (e.g., vegetables) contaminated with *T. martis* eggs. It is conceivable that taeniid eggs can adhere to soil and grass when spread, e.g., by rainfall, from the feces of free-ranging martens [1,5]. Another possible transmission pathway is the direct uptake of contaminated feces by the marmosets during foraging activities, especially when searching for fruit stones. This route of infection has been proposed in the case of the Tonkean macaque with *T. martis* cysticercosis from a French primate center with access to a fenced park-like facility [1]. In fact, all cases of cysticercosis in non-human primates occurred in animals from zoo collections or primate centers with access to outdoor enclosures [1,2,5,13]. The fact that infection with parasite eggs may occur by ingestion of contaminated fresh vegetables emphasizes the zoonotic potential of this parasite [13].

In non-human primates, *T. martis* cysticercosis has so far only been observed in the abdominal and/or thoracic cavities, usually involving the mesentery and/or serosal surfaces [2]. In humans, there are two reports of ocular manifestations [7,8] and three cases of neurocysticercosis [6,11,12], aside from two peritoneal infections [9,10].

Regarding the etiological diagnosis of cysticercosis, morphological characteristics of the cestode larvae often allow a tentative diagnosis on the species level. In histological sections, cysticerci present as rather small (5–15 mm) smooth or warty bladders with an eosinophilic tegument covered by microvilli-like structures (microtriches), a fibrillary parenchyma containing calcareous corpuscles, and one invaginated scolex (monocephalic) with a hooked rostellum and muscular suckers [18,19]. Species-specific differences in the number, shape, and size of rostellar hooks are the most important diagnostic criteria [1,18]. In addition, some metacestodes exhibit unique morphological features, e.g., exo- or endogenous budding in cysticerci of *T. crassiceps*, which has also been described in non-human primates [18,20]. However, due to interspecies overlap of morphological characteristics or the absence of differentiating structures, e.g., in damaged cysticerci, molecular analysis is essential for the confirmation of the cestode species [2].

In the common marmoset, cysticerci were frequently degenerated and scolex parts could not be observed. Thus, species identification was only possible using molecular techniques. Interestingly, a conspecific from the same marmoset husbandry was also diagnosed with a cestode larval infection. However, in contrast to the marmoset with *T. martis* cysticercosis, this other animal harbored metacestode tissue with the typical histological appearance of alveolar echinococcosis, a larval tape worm infection, which has been reported in several non-human primate species [21]. The larval stage of *Echinococcus multilocularis* is a modified cysticercus with multiple protoscolices (polycephalic). In histological sections, larval cysts are lined by an inner germinal epithelium and a prominent outer PAS (periodic acid Schiff)-positive lamellar hyaline membrane. Brood capsules bud from the inner germinal layer and contain variable numbers of invaginated protoscolices [22]. The fact that two animals from the same husbandry developed cestode larval infections is indicative of their increased exposure to egg-containing feces, either by food and soil contamination or by direct uptake of contaminated feces in contact zones between intermediate and definitive hosts.

In conclusion, a cestode larval infection has to be considered as a differential diagnosis for cystic masses in body cavities in marmosets and other non-human primates. In cysticercosis, molecular testing is obligatory for the exact determination of the causative cestode species [2]. Exact classification of the respective parasite forms an essential prerequisite for detecting previously unknown susceptible aberrant intermediate hosts, to evaluate geographic distribution patterns and prevalences, to understand general pathogen biology and life cycles, and to assess the risk of exposure for both humans and animals.

## Data Availability

The raw data supporting the conclusions of this article will be made available by the authors on request.

## References

[1] Brunet J., Pesson B., Chermette R., Regnard P., Grimm F., Deplazes P., Ferreira X., Sabou M., Pfaff A.W., Abou-Bacar A. (2014). First case of peritoneal cysticercosis in a non-human primate host (*Macaca tonkeana*) due to *Taenia martis*. Parasit. Vectors.

[2] Danière C., Callait-Cardinal M.P., Grenouillet F., Lemberger K., Quintard B. (2024). *Taenia martis* in an Alaotran gentle lemur (*Hapale-mur alaotrensis*): The importance of molecular identification. Vet. Rec. Case Rep..

[3] Prokopic J. (1970). Some notes on the distribution and life history of the cestode *Taenia martis* (Zeder, 1803). Helminthologia.

[4] Loos-Frank B., Zeyhle E. (1982). The intestinal helminths of the red fox and some other carnivores in southwest Germany. Z. Parasitenkd..

[5] Peters M., Mormann S., Gies N., Rentería-Solís Z. (2023). *Taenia martis* in a white-headed lemur (*Eulemur albifrons*) from a zoological park in North Rhine-Westphalia, Germany. Vet. Parasitol. Reg. Stud. Rep..

[6] Brunet J., Benoilid A., Kremer S., Dalvit C., Lefebvre N., Hansmann Y., Chenard M.P., Mathieu B., Grimm F., Deplazes P. (2015). First case of human cerebral *Taenia martis* cysticercosis. J. Clin. Microbiol..

[7] Eberwein P., Haeupler A., Kuepper F., Wagner D., Kern W.V., Muntau B., Racz P., Agostini H., Poppert S. (2013). Human infection with marten tapeworm. Emerg. Infect. Dis..

[8] Koch T., Schoen C., Muntau B., Addo M., Ostertag H., Wiechens B., Tappe D. (2016). Molecular Diagnosis of Human *Taenia martis* eye infection. Am. J. Trop. Med. Hyg..

[9] Mueller A., Förch G., Zustin J., Muntau B., Schuldt G., Tappe D. (2020). Case Report: Molecular identification of larval *Taenia martis* infection in the Pouch of Douglas. Am. J. Trop. Med. Hyg..

[10] Rudelius M., Brehm K., Poelcher M., Spinner C., Rosenwald A., da Costa C.P. (2017). First case of human peritoneal cysticercosis mimicking peritoneal carcinosis: Necessity of laparoscopy and histologic assessment for the correct diagnosis. JMM Case Rep..

[11] Steinsiepe V.K., Ruf M.T., Rossi M., Fricker-Feer C., Kolenc D., Buser B.S., Concu M., Neumayr A., Schneider U.C. (2023). Human *Taenia martis* neurocysticercosis, Switzerland. Emerg. Infect. Dis..

[12] Eggink H., Maas M., van den Brand J.M.A., Dekker J., Franssen F., Hoving E.W., Kortbeek L.M., Kranendonk M.E.G., Meiners L.C., Rittscher A.E. (2024). *Taenia martis* neurocysticercosis-like lesion in child, associated with local source, the Netherlands. Emerg. Infect. Dis..

[13] De Liberato C., Berrilli F., Meoli R., Friedrich K.G., Di Cerbo P., Cocumelli C., Eleni C. (2014). Fatal infection with *Taenia martis* metacestodes in a ring-tailed lemur (*Lemur catta*) living in an Italian zoological garden. Parasitol. Int..

[14] Nguyen N., Fashing P.J., Boyd D.A., Barry T.S., Burke R.J., Goodale C.B., Jones S.C., Kerby J.T., Kellogg B.S., Lee L.M. (2015). Fitness impacts of tapeworm parasitism on wild gelada monkeys at Guassa, Ethiopia. Am. J. Primatol..

[15] Gouy M., Guindon S., Gascuel O. (2010). SeaView version 4: A multiplatform graphical user interface for sequence alignment and phylogenetic tree building. Mol. Biol. Evol..

[16] Segovia J.M., Torres J., Miquel J., Sospedra E., Guerrero R., Feliu C. (2007). Analysis of helminth communities of the pine marten, *Martes martes*, in Spain: Mainland and insular data. Acta Parasit..

[17] Millán J., Ferroglio E. (2001). Helminth parasites in stone martens (*Martes foina*) from Italy. Eur. J. Wildl. Res..

[18] Deplazes P., Eichenberger R.M., Grimm F. (2019). Wildlife-transmitted *Taenia* and *Versteria* cysticercosis and coenurosis in humans and other primates. Int. J. Parasitol. Parasites Wildl..

[19] Chervy L. (2002). The terminology of larval cestodes or metacestodes. Syst. Parasitol..

[20] Bleyer M., Risch T., Roos C., Kaup F.J., Mätz-Rensing K. (2018). *Taenia crassiceps cysticercosis* in a Nilgiri langur (*Semnopithecus johnii*). J. Zoo Wildl. Med..

[21] Yamano K., Kouguchi H., Uraguchi K., Mukai T., Shibata C., Yamamoto H., Takaesu N., Ito M., Makino Y., Takiguchi M. (2014). First detection of *Echinococcus multilocularis* infection in two species of nonhuman primates raised in a zoo: A fatal case in *Cercopithecus diana* and a strongly suspected case of spontaneous recovery in *Macaca nigra*. Parasitol. Int..

[22] Lampe K. (2013). Untersuchungen zur Diagnostik und Prophylaxe der alveolären Echinokokkose bei Makaken (German).

